# Wavelength de-multiplexing metasurface hologram

**DOI:** 10.1038/srep35657

**Published:** 2016-10-18

**Authors:** Bo Wang, Baogang Quan, Jingwen He, Zhenwei Xie, Xinke Wang, Junjie Li, Qiang Kan, Yan Zhang

**Affiliations:** 1 Department of Physics, Capital Normal University, Beijing Key Laboratory of Metamaterials and Devices, Key Laboratory of Terahertz Optoelectronics, Ministry of Education, and Beijing Advanced Innovation Center for Imaging Technology, Beijing, 100048, China; 2 Beijing National Laboratory for Condensed Matter Physics, Institute of Physics, Chinese Academy of Sciences, Beijing 100190, China; 3 Department of Physics, Harbin Institute of Technology, Harbin, 150001, China; 4 Key Laboratory of Semiconductor Materials Science, Institute of Semiconductors, Chinese Academy of Science, Beijing 100083, China

## Abstract

A wavelength de-multiplexing metasurface hologram composed of subwavelength metallic antennas is designed and demonstrated experimentally in the terahertz (THz) regime. Different character patterns are generated at the separated working frequencies 0.50 THz and 0.63 THz which determine a narrow frequency bandwidth of 130 GHz. The two working frequencies are around the central resonance frequency of the antennas where antennas behave strong wavefront modulation. Each antenna is fully utilized to control the wavefront of the metasurface at different frequencies by an optimization algorithm. The results demonstrate a candidate way to design multi-colors optical display elements.

Based on the electromagnetic resonance of subwavelength plasmonic antennas, metasurface has been developed as ultra-thin metallic wavefront modulators in the recent years^1^,^2^,^3^,^4^,^5^,^6^,^7^,^8^. Not like conventional optical elements composed of dielectric medium which need enough thickness to accumulate gradual phase changes along the optical path, the plasmonic resonance of metasurface can provide 2π phase delay of cross-polarization conversion light at a thickness much smaller than the wavelength^9^. From microwave^10^ to infrared^11^ and visible regime^12^, many antenna structures are proposed as the wavefront modulator unit such as V-shaped^3^,^6^,^13^, bar shaped^14^,^15^,^16^, C-shaped^17^,^18^ and their Babinet-inverted shape^19^. Various functional metasurface devices such as vortex^2^,^20^,^21^, focusing lens^3^,^15^,^16^,^22^, laser beam shaper^14^ and holograms^12^,^13^,^22^,^23^,^24^ are studied to show the miniaturization and integration of diffraction optical elements. Wavelength and polarization de-multiplexing metasurface devices are also reported recently^25^,^26^.

Many of the proposed planar metasurfaces are broadband. The metasurface of cylindrical, spherical and cone lens can focus the incident planewave in a broadband regime which only exhibit focus spot shifting with the wavelength^3^,^19^,^27^. The metasurface of beam deflector can bend the reflected or refracted beam with different angle for wavelength in a broadband regime^18^. The metasurface of quarter wave plate is also broadband^4^. This is mainly due to broad bandwidth of plasmonic resonance mode in the subwavelength antennas. This is an obstacle for the development of wavelength de-multiplexing metasurface which working at separated nearby frequencies. Recently, by spatial arrangement of aluminum nanorod with surface plasmon resonances in red, green and blue lights, a multi-color metasurface hologram^23^ in visible regime was achieved which reconstruct character patterns in the separated reflective angle for each working wavelength. In the recent progress, wavelength demultiplexing using spatial arrangement and nonlinear harmonics has also been reported^25^,^26^. However, the frequency is limited to the fundamental and its second harmonics.

In this paper, we proposed a new concept for designing multi-color metasurface hologram which fully utilizes the wavefront modulation capacity of each antenna at different working frequencies. The rebuilt character patterns for different frequency can overlap in the transmission zone, thus three primary colors can be used to generate a colorful image. A dual-color THz metasurface hologram working at 0.50 THz and 0.63 THz based on C-shaped metallic antenna arrays is designed, fabricated and measured to verify this concept. Experiment results demonstrated the theoretical expectation. This method will provide more spatial bandwidth product for a better hologram designing and benefit the multi-color synthesis at the same observation position.

## Principle

**Figure 1:**
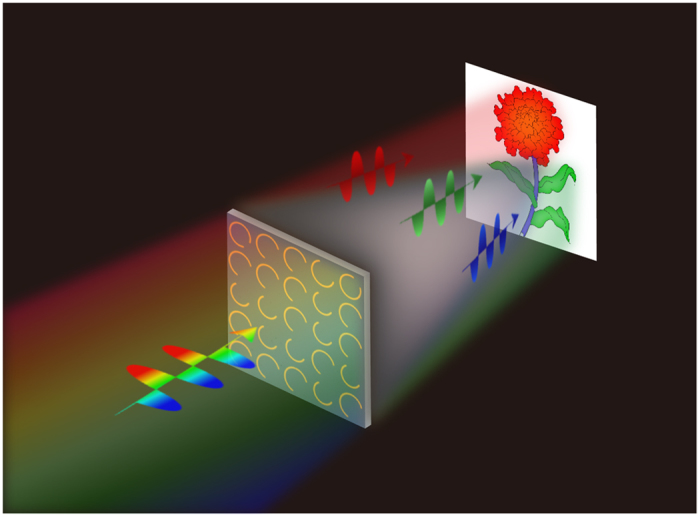
Diagrammatic sketch of a wavelength de-multiplexing metasurface hologram. The incident light is polarized horizontally and the detected transmission light is polarized perpendicularly.

The basic ideal for multi-color metasurface hologram is presented in Fig. 1. The metasurface device is composed of predesigned antennas. When the device is illuminated with desired multi-wavelength light, the light with different frequency will be modulated by each antenna. On the preset plane, the light with same frequency will interfere and generate the desired pattern. The pattern generated by different frequency can overlap with each other, thus a colorful image can be generated on the preset plane. In order to demonstrate this concept, a metasurface device in the THz range is designed, fabricated, and characterized. The device is composed of 128*128 metallic (gold) antennas deposited on a high resistive silicon substrate. Each antenna contributs to the wavefront modulation of metasurface at the preset working frequencies. When the metasurface is illuminated by the linearly polarized plane wave from the substrate side, the image can be reconstructed by the transmitted cross-polarized light. The observation plane for 0.50 THz and 0.63 THz is the same which locates at a distance 5.0 mm from the structure plane of metasurface. The character image “C” and “N” will be reconstructed for 0.50 THz and 0.63 THz, respectively. Two character images overlap at the observation plane for their working frequency simultaneously.

**Figure 2:**
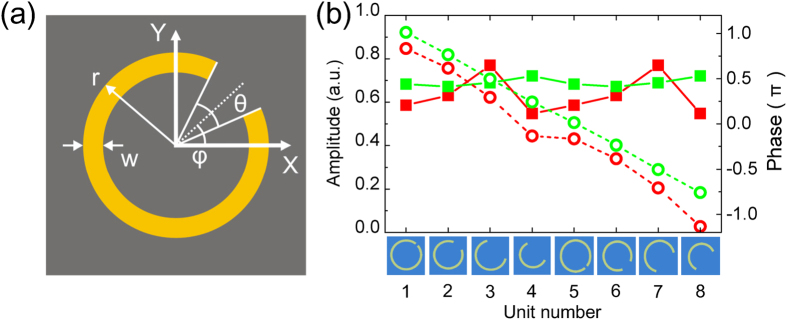
(**a**) Geometry of a C-shaped antenna. (**b**) Amplitude and phase modulations of selected antenna units. The solid lines are uniformed to the amplitude of incident light, the dashed lines are phase of transmited light. The red and green lines correspond to the modulations for 0.50 THz and 0.63 THz, respectively.

The same C-shaped subwavelength metallic antennas proposed by Zhang *et al*. in the THz regime^18^, as shown in Fig. 2(a), are adopted as dual-color wavefront modulator units. The period of C-shaped antennas is 80 μm, which is about 1/5 smaller than the working wavelength. At 0.63 THz, the antennas from No. 1 to No. 8 provide pure phase modulation with total phase range of 2π and a phase step of π/4, as shown by green dot curve in Fig. 2(b). But for 0.50 THz, the biggest deviation of amplitude modulation of antennas (red solid curve) is about 20% and phase step is not always π/4 for two neighbor antennas, especially for No. 4 and No. 5 (red dot curve). Thus it’s better to consider both amplitude and phase modulation of the hologram for achieving a good reconstruction quality.

## Results

**Figure 3:**
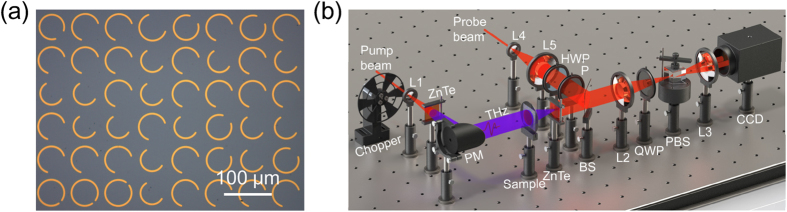
(**a**) Optical image of part of sample (part of view) and (**b**) THz focal plane imaging system for device characterization.

The dual-color THz metasurface hologram is designed by the simulated annealing (SA) algorithm (see Methods) and fabricated by the ultraviolet lithography. The antenna arrays are transferred to the gold film which is deposited on the high resistive silicon substrate. The thickness of gold film and silicon substrate is 100 nm and 350 μm, respectively. The metasurface is composed of 128 × 128 pixels with total size of 10.240 mm × 10.240 mm, as shown in Fig. 3(a). The propagation distance between the observation plane and exit plane of metasurface device is 5.0 mm.

The rebuilt character patterns of THz metasurface hologram at the observation plane are recorded using a THz focal plane imaging system^21^,^22^ based on electro-optics sampling method, see Fig. 3(b). The THz pulse is generated by a femtosecond laser beam with the central wavelength of 800 nm exciting on a ZnTe crystal. The parabolic mirror (PM) collects and collimates the THz radiation. Another ZnTe crystal is used to detect the transmitted cross-polarization wave located on the observation plane (see Methods).

**Figure 4:**
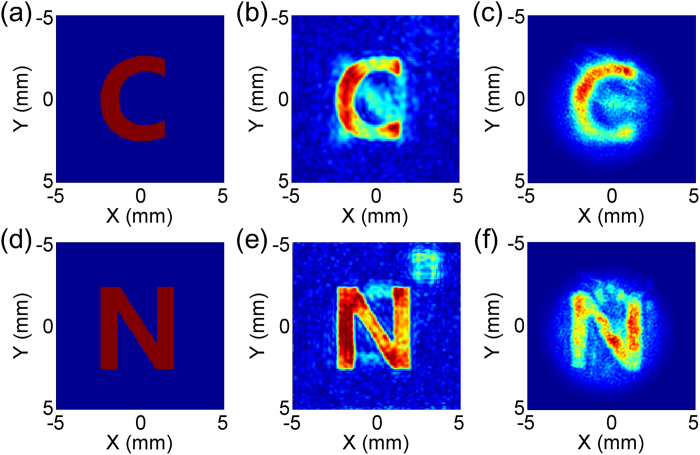
(**a**–**c**) are the objective image, design result and measurement result of the sample at 0.50 THz. (**d**–**f**) are the objective image, design result and measurement result of the sample at 0.63 THz.

The recorded hologram patterns at 0.50 THz and 0.63 THz are shown in Fig. 4(c,f), respectively. The desired objective images Fig. 4(a,d) and the design results Fig. 4(b,e) obtained by the simulated annealing algorithm are also displayed for comparison. The x- and y- axis are corresponding to the horizontal and perpendicular polarization directions of light. The optical axis is located at (x = 0, y = 0) which centered at the detection area. From the designed results shown in Fig. 4(b,e), it can be seen that the rebuilt character patterns are overlapped around the optical axis with slight cross-talks for each working frequencies. The measured “C” and “N” patterns in Fig. 4(c,f) agree well with the desired patterns about the character shape, location and size at 0.50 THz and 0.63 THz. Efficiency is always an important issue for practical applications^28^. The cross-polarization conversion efficiency of the device is above 10%, measured with a THz time domain spectroscopy at the working frequencies.

**Figure 5:**
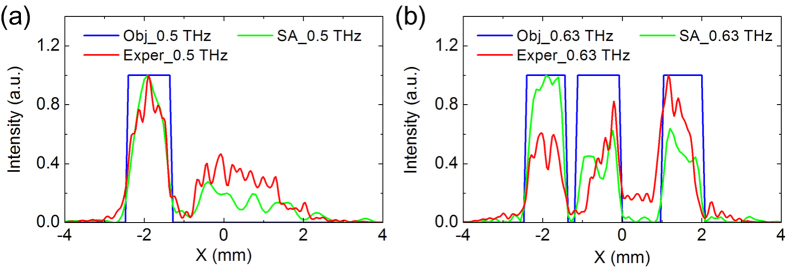
(**a**,**b**) are the intensity distributions of objective image, design result and measurement result of the sample alone the line y = 0 at 0.5 THz and 0.63 THz, respectively.

In Fig. 5, the intensities of object images, design results, and recorded images along the straight line y = 0 are presented for comparison. In Fig. 5(a), one peak between x = −2.5 mm and x = 0 mm indicates the character “C” rebuilt at working frequency 0.50 THz. In Fig. 5(b), three peaks are observed due to the character “N” rebuilt at 0.63 THz. The measured results behave lower signal noise ration than the design results obtained by the simulated annealing algorithm at both working frequencies while the main features can be distinguished by bare eyes.

## Discussion

A narrow-band wavelength de-multiplexing metasurface hologram is proposed. A THz dual-color synthesis device based on the wavefront modulation of metallic subwavelength C-shaped antennas is demonstrated. The character patterns are rebuilt by the transmited cross-polarization light at the same observation plane. The metasurface is designed by the SA algorithm to fully utilize the spatial bandwidth product in the limited total size while each antenna performs strong amplitude and phase modulations at the two nearby working frequencies. It is expected this method can be extended to visible range to achieve RGB hologram.

## Methods

The amplitude and phase modulations of the metallic antennas at different frequency are calculated by a commercial software package FDTD Solution based on the finite-difference time-domain method. The metasurface consisted of antenna arrays is optimized by the SA algorithm. The SA algorithm^29^ is not parallelized and the computation cost becomes huge when the number of total pixel increases. We adopted two ways to accelerate the convergence speed. One is the optimized annealing temperature curve. An exponential temperature curve is selected as *t*(*k*) = *t*_0_ × *α*^*k*^, where *t*_0_ is the initial temperature, *k* is iteration number and *α* is the temperature ratio which is set to 0.98 in the optimization. The other is difference calculation method of Fresnel diffraction. In the iterations, we evaluate each pixel one by one. The discrete form of Fresnel diffraction integral to compute the observation-plane optical field *U*_2_(*r*, *c*) from knowledge of the source-plane optical field *U*_1_(*j*, *k*) is expressed as:


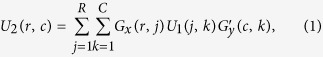


where *G*_*x*_(*r*, *j*) and *G*_*y*_(*c*, *k*) are the Fresnel transform matrix,


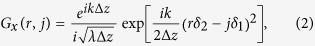



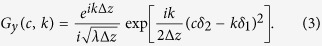


In each iteration, a pixel of source-plane optical field *U*_1_(*j*, *k*) is changed from one antenna to another antenna with the increment Δ*U*_*ξ*,*η*_,


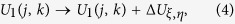


then the observation-plane optical field *U*_2_(*r*, *c*) is changed to


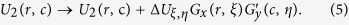


Noted only at one pixel Δ*U*_*ξ*,*η*_ is nonzero, thus the computation is reduced from calculation of product of three matrix in Eq. (1) to the product of two one-dimensional vectors *G*_*x*_(*r*, *ξ*) and *G*_*y*_(*c*, *η*) in Eq. (5). Compared with other methods to calculate the wave propagation, this approach is faster at the cost of accuracy. However, experiment results demonstrate this approach is acceptable.

### Measurement method

A THz focal plane imaging system is adopted to character the performances of the device^30^. A laser beam with central wavelength 800 nm, repeated frequency 1 kHz, pulse width 100 fs and average power 800 mW is divided to pump beam and probe beam by a beam splitter. The pump beam is incident on a <110> ZnTe crystal with 3 mm thickness to generate THz radiation. Then the THz radiation is collected and collimated by a 90 degree off-axis metallic parabolic mirror with 150 mm focal length. Thus the THz beam is converted to a linearly polarized plane wave and illuminates on the sample normally. The probe beam is expanded to about 15 mm diameter by a combination of concave and convex lens. Then a 50/50 beam splitter is used to reflect the probe beam to electro-optical detecting crystal (the other <110> ZnTe with 3 mm thickness). Finally, the probe beam modulated by the THz radiation in the crystal is reflected by the back surface of ZnTe crystal and recorded by an imaging module including a quarter wave plate, two lenses, a Wollaston prism and a CY-DB1300A CCD. The CCD is synchronized with the chopper in the pump beam to acquire the THz image with the dynamics subtraction technique. The mechanics delay line is adopted to scan a total 10 ps THz temporal images with a step of 20 μm. To enhance the signal to noise ratio, 100 frames are recorded and averaged at each scan position.

In the experimental, the co-polarization component is filtered out by the polarization dependent detection method^30^. In the THz focal plane imaging system used in this work, a <110> ZnTe is used to detect THz wave. The horizontal component of the THz electric field can be measured when the probe polarization is parallel to the <001> axis of the crystal, and the half of the vertical component can be measured when the angle between the probe polarization and the <001> axis is 45°. The probe laser beam is linearly polarized and the polarization state can be rotated by a half wave plate (HWP), thus two components of the THz wave can be achieved.

## Additional Information

**How to cite this article**: Wang, B. *et al*. Wavelength de-multiplexing metasurface hologram. *Sci. Rep.* **6**, 35657; doi: 10.1038/srep35657 (2016).
